# MXene-Embedded PEDOT:PSS Hole-Transport Material for Lead-Free Perovskite Solar Cells

**DOI:** 10.1021/acsaem.3c02928

**Published:** 2024-08-23

**Authors:** JinKiong Ling, Daniele T. Cuzzupè, Muhammad Faraz Ud Din, Anastasiia Stepura, Tom Burgard, Yekitwork Abebe Temitmie, Eva Majkova, Maria Omastova, Rajan Jose, Lukas Schmidt-Mende, Azhar Fakharuddin

**Affiliations:** ^§^Department of Physics and ^∥^Zunkunftskolleg, University of Konstanz, 78457 Konstanz, Germany; ⊥ Institute of Photovoltaic, 18 Boulevard Thomas Gobert, 91120 Palaiseau, France; ^◆^Institute of Physics and ^¶^Polymer Institute, Slovak Academy of Sciences, 84541 Bratislava 45, Slovak Republic; ^∇^Center for Advanced Intelligent Materials and ^○^Faculty of Industrial Sciences and Technology, Universiti Malaysia Pahang, 26300 Pahang, Malaysia; 5 Department of Physics, University of Bahir Dar, 6000 Bahir Dar, Ethiopia

**Keywords:** 2D materials, tin perovskite, quasi-2D perovskites, phenylethylamine cation (PEAI), hole-transporting materials, perovskite solar cells

## Abstract

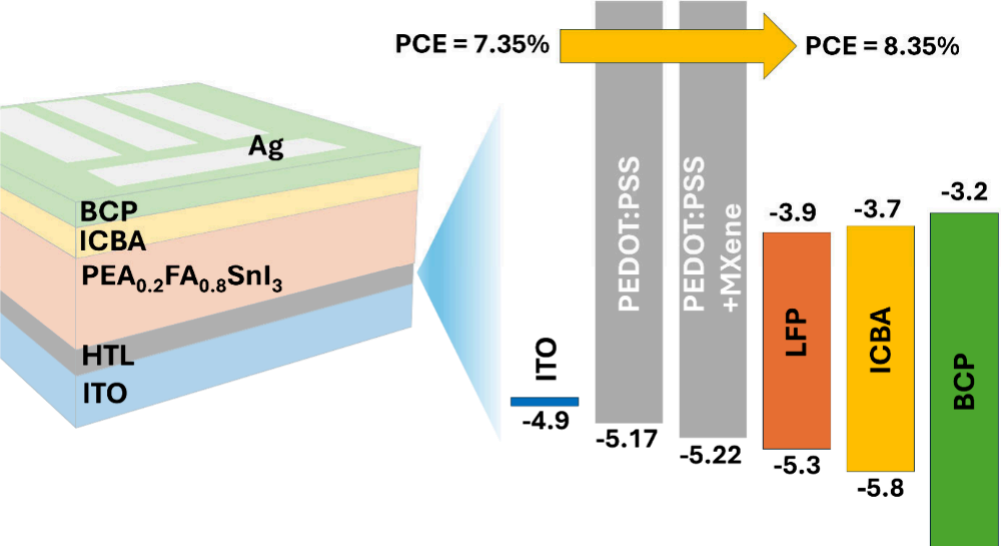

Improving the energy alignment between charge-transport layers and the perovskite is crucial for further enhancing the photovoltaic performance of tin-based perovskite solar cells (PSCs). Herein, the role of Ti_3_C_2_T_*x*_ MXene in a poly(3,4-ethylenedioxythiophene):poly(styrenesulfonate) (PEDOT:PSS) hole transport layer (HTL) on the photovoltaic properties of PSCs is investigated as a function of its concentration. An improved perovskite film formation with reduced pinhole density and a more uniform contact potential difference is noted when MXene is embedded in the PEDOT:PSS HTL. The work function of the HTL is increased according to photoelectronic measurements, leading to a favorable energy alignment with the HOMO of PEA_0.2_FA_0.8_SnI_3_ perovskite. PSCs fabricated using a MXene-embedded PEDOT:PSS HTL delivered a power conversion efficiency (PCE) of 8.35% compared to 7.35% from the pristine counterpart, while retaining ∼90% of its initial PCE after 450 h of storage in a N_2_ atmosphere.

The requirement of low lead content in electronic appliances from regulatory bodies around the world has triggered tremendous attention on developing lead-free perovskites (LFPs) with a photovoltaic performance comparable to that of their lead-based counterparts.^1−3^ To date, tin-based halide perovskites are the most promising alternative among lead-based perovskites, with certified power conversion efficiency (PCE) reaching ∼14% in an inverted p–i–n device architecture.^4−8^ Nevertheless, the energy-level misalignments between the LFP light absorber and its transport layers resulted in a large open-circuit voltage (*V*_OC_) deficit.^9,10^ Additionally, the rapid crystallization of tin halide perovskites causes inhomogeneous film formation, limiting the PCE of perovskite solar cells (PSCs).^11−13^ With tin halide perovskite showing an optical absorption coefficient and excellent charge mobility identical with those of their lead-based counterparts,^14^ matching the energy level between the charge-selective layers and the perovskite could be a critical proposition to further improve the performance of tin-based PSCs. To date, state-of-the-art tin-based PSCs employ the p–i–n (or inverted) device structure,^5,6^ with tin halide perovskite deposited on top of a poly(3,4-ethylenedioxythiophene):poly(styrenesulfonate) (PEDOT:PSS), a hole-transport material with high transparency, good perovskite solution wettability, and ease of deposition.^15^ However, PEDOT:PSS suffers from inferior electrical conductivity, inferior electron blocking capability, and poor energy matching with tin halide perovskites, severely limiting the photovoltaic performance of the tin-based PSCs.^16^ Tuning the electronic properties of PEDOT:PSS to enhance the photovoltaic performance by embedding additives could effectively address these concerns.^17−22^ Utilizing two-dimensional MXenes as additives to improve the photovoltaic performance of organic solar cells and PSCs has slowly gained traction.

MXenes are denoted by M_*n*+1_X_*n*_T_*x*_, where M represents any transition metal (such as Ti, V, Mo, Ta, Hf, Ce, etc.), while X represents carbon and/or nitrogen. Ti_3_C_2_T_*x*_ possesses superior electrical conductivity (∼104 S cm^–1^), high carrier density (∼3.8 × 10^22^ cm^–3^), and high charge mobility (∼300 cm^2^ V^–1^ s^–1^).^23−25^ The surface functional groups of MXenes, i.e., Ti_3_C_2_T_*x*_ (where T_*x*_ can be -F, =O, or -OH), could slow down the perovskite crystallization process,^26^ leading to improved film formation and higher PCE.^27^ The use of Ti_3_C_2_T_*x*_ as an additive (in charge-transport layers as well as in the perovskite absorbers) or as an interlayer was previously reported to passivate the surface trap states,^28^ significantly reducing trap-assisted charge recombination and improving the photovoltaic performance.^29^ However, completely replacing the charge-transport layer with MXenes was reported to not offer any significant improvement and concluded that the MXenes serve better as additives for the components in lead-based PSCs.^30^ An increase in the hole mobility (from ∼3.39 × 10^–4^ to ∼5.76 × 10^–4^ cm^2^ V^–1^ s^–1^) was reported in PEDOT:PSS through Mo_1.33_C integration, leading to a higher short-circuit current (*J*_SC_) in PEDOT:PSS/PTB7-Th:PC_71_BM organic solar cells.^31^ In another example, embedding WO_*x*_ nanoparticles in the PEDOT:PSS hole-transport layer (HTL) lead to an improved device performance in organic solar cells due to better alignment of the work function and balanced electron–hole extraction.^32^ The WO_*x*_-embedded PEDOT:PSS HTL was also tested for lead-based (MAPbI_3_) PSCs, leading to a higher PCE due to improved charge-transport properties.^33^ Despite the growing interest in utilizing two-dimensional MXenes as additives to improve the photovoltaic performance of lead-based PSCs, to the best of our knowledge, their application in tin-based PSCs has not been investigated. Coupled with the efficient electron blocking characteristics of Ti_3_C_2_T_*x*_,^34^ embedding Ti_3_C_2_T_*x*_ shows prospects in enhancing the photovoltaic performance of tin-based PSCs by tuning the electronic properties of PEDOT:PSS.

In this work, Ti_3_C_2_T_*x*_ MXenes were prepared by delaminating the Ti_3_AlC_2_ MAX phase, as demonstrated in Figure S1 (Figure S2 shows the delaminated MXenes solution). More details on the synthesis of Ti_3_C_2_T_*x*_ can be found in the Supporting Information. X-ray diffraction (XRD) analysis in Figure S3 shows distinctive diffraction peaks corresponding to that of the delaminated MXenes layer, with small amounts of the unreacted MAX phase at 2θ ∼ 10°. The flakelike morphology of the delaminated Ti_3_C_2_T_*x*_ can be observed through transmission electron microscopy (Figure S4), with the flake width in the range of 2 μm. The high transparency of the Ti_3_C_2_T_*x*_ flakes indicates that the delaminated MXenes have low thickness. No observation can be made regarding the stacking layer at the peripheral of the flake, showing that the delaminated Ti_3_C_2_T_*x*_ flakes are mostly single-layered.^24,35^ The surface components of the MXenes (T as in Ti_3_C_2_T_*x*_) were analyzed using X-ray photoelectron spectroscopy (XPS), where a mixture of -F and -O surface elements were detected (Figure S5 and Table S1). These surface elements will affect the work function of Ti_3_C_2_T_*x*_ and PEDOT:PSS modified with MXenes.^36^

The effect of Ti_3_C_2_T_*x*_ incorporation on the local work function of PEDOT:PSS was studied by using Kevin probe force microscopy (KPFM). Increasing the concentration of Ti_3_C_2_T_*x*_ was observed to deepen the work function of PEDOT:PSS, as shown in Figure 1a. This observation was in line with the data acquired from photoelectron spectroscopy in air (PESA) in Figure S6 and summarized in Figure 1b. The deep work function of pristine Ti_3_C_2_T_*x*_ at −5.7 eV could lower the work function of PEDOT:PSS from −5.17 to −5.22 eV after the incorporation of small amounts of MXene. Such a modification could align the work function of PEDOT:PSS closer to that of tin-based perovskite, where improved performance can be anticipated. Notably, the inclusion of Ti_3_C_2_T_*x*_ in PEDOT:PSS also affects the local contact potential difference distribution (ΔCPD), as shown in Figure S7. The Ti_3_C_2_T_*x*_-modified HTLs generally showed a smaller variation in CPD than the PEDOT:PSS counterpart (Figure S8), which is preferable for efficient charge transport.^37,38^ The highest ΔCPD is measured for the pristine PEDOT:PSS HTL, which could be attributed to the inhomogeneous morphology and the presence of pinholes in the film. Increasing the Ti_3_C_2_T_*x*_ concentration beyond 16 μg mL^–1^ would lead to clustering of the MXene flakes in the HTL, which results in significant local variation in the work function (Figure S9; MXene flakes induce a work function dip of over 700 meV). Such clustering could exacerbate the energy-level misalignment between the HTL and tin-based perovskite, disrupting hole extraction, and lead to a reduction in the photocurrent.

**Figure 1:**
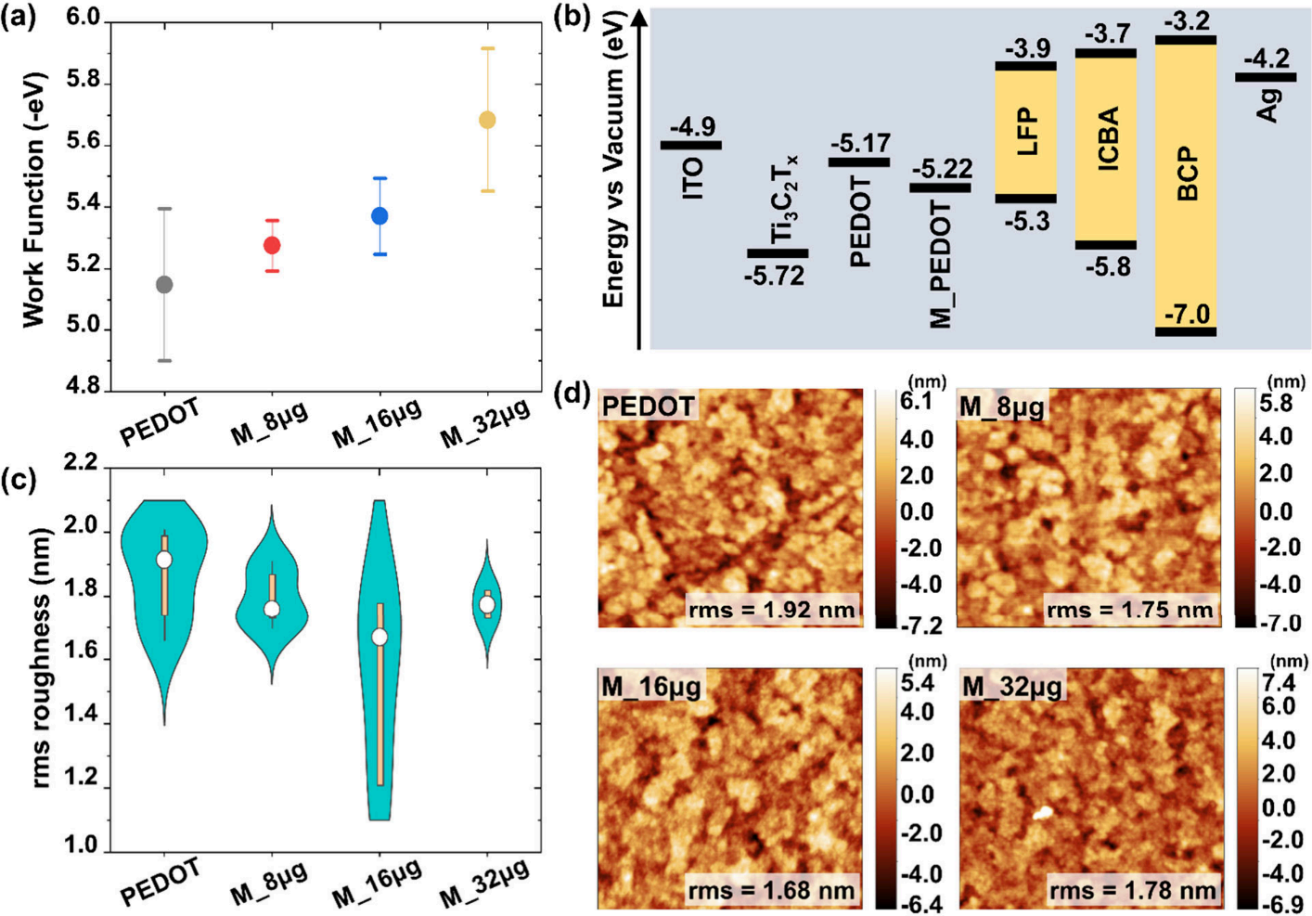
(a) Statistical distribution of the work function measured over at least four spots on two different samples for each sample type, obtained from KPFM. (b) Work function of the HTLs measured using PESA. (c) Statistical distribution of root-mean-square roughness measured over at least four spots on two different samples for each sample type, obtained from AFM. (d) AFM morphology of the synthesized HTLs.

To gain insights into morphological differences, atomic force microscopy (AFM) measurements were performed for all HTLs under controlled humidity (relative humidity below 3%). All samples show nearly identical morphology, although a reduction in the root mean square (rms) roughness is noted upon Ti_3_C_2_T_*x*_ incorporation (Figure 1c,d). Increasing the Ti_3_C_2_T_*x*_ concentration beyond 16 μg mL^–1^ leads to an increase in the rms roughness, which could be unfavorable for quality perovskite formation, and it could lead to interfacial traps. Scanning electron microscopy (SEM) was used to observe the morphology of the tin-based perovskite films deposited on the HTLs. Pinholes can be clearly observed in the pristine PEDOT:PSS and in the M_32 μg samples (Figure 2a), indicating poor wetting of PEDOT:PSS by the perovskite precursor solution. The pinholes observed in M_32 μg could be the result of Ti_3_C_2_T_*x*_ flake aggregation, which would negatively impact these layers’ wettability and result in inhomogeneous perovskite film formation. Cross-sectional SEM of the perovskite coating on the reference and on the M_8 μg HTL sample (Figure S10) shows identical perovskite film thickness (with a thickness of around 160 nm). The distribution curves of the tin-based perovskite grain size measured from 100 grains (Figure S11) demonstrated the capability of Ti_3_C_2_T_*x*_-modified PEDOT:PSS in promoting grain growth at a concentration of 8 μg mL^–1^ (average grain size of 164.2 nm), while increasing the concentration beyond 16 μg mL^–1^ reduces the average grain size to 141.21 nm, further down to 140.7 nm for 32 μg mL^–1^, and 109.5 nm for pristine PEDOT:PSS. Evidently, the inclusion of Ti_3_C_2_T_*x*_ could reduce the pinhole density and enhance the film quality of the tin-based perovskite films, which can be beneficial in enhancing the photovoltaic performance.

**Figure 2:**
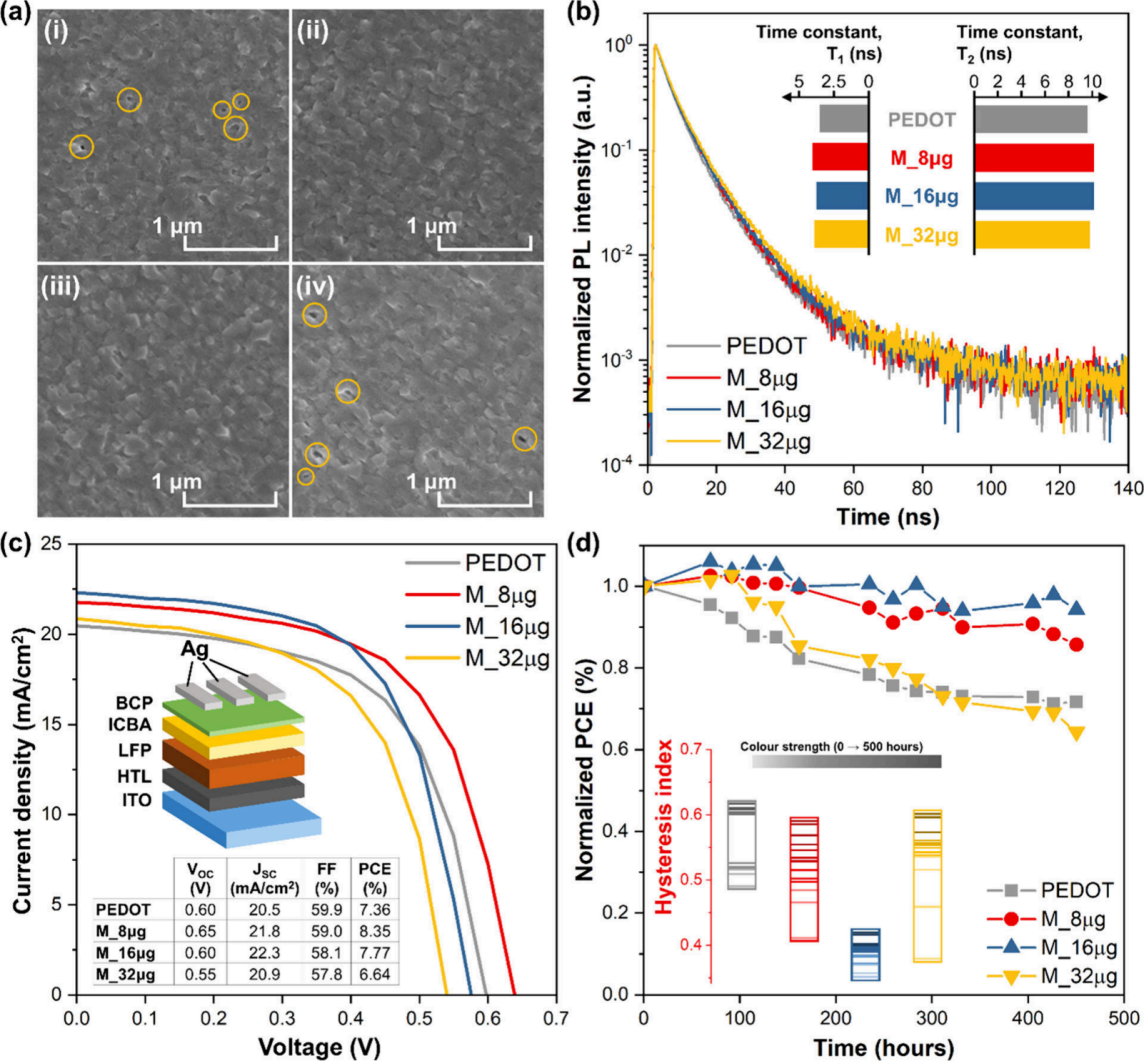
(a) SEM morphological observation of a tin-based perovskite film deposited on top of (i) PEDOT:PSS, (ii) M_8 μg, (iii) M_16 μg, and (iv) M_32 μg, respectively. (b) TRPL spectra for the respective samples. The inset shows the time constants extracted through fitting of the TRPL spectra with biexponential functions. (c) *J*–*V* curves of the champion devices during a forward scan. The inset shows the inverted p–i–n device structure tested and their respective photovoltaic parameters. (d) PCE stability testing for 450 h. The inset shows the hysteresis index of the devices throughout the stability testing.

The optoelectronic properties of the tin-based perovskite deposited on the HTLs were studied using steady-state photoluminescence (PL) and time-resolved photoluminescence (TRPL). The PL spectra for all samples in Figure S12 show emission peaks centered at 873 nm, equivalent to an optical band gap of 1.42 eV. No shifting in the PL peaks were observed after the modification of PEDOT:PSS, indicating that the inclusion of Ti_3_C_2_T_*x*_ in PEDOT:PSS does not alter the chemical composition of the tin-based perovskite. The TRPL spectra of the samples (Figure 2c) show biexponential decays with fast (τ_1_) and slow (τ_2_) decay components. These decays correspond to the interfacial charge extraction and trap-assisted recombination (τ_1_) and bimolecular recombination (τ_2_), respectively.^39^ We note that the maximum carrier lifetime could be achieved with the addition of 8 μg mL^–1^ Ti_3_C_2_T_*x*_. The average carrier lifetime was calculated from τ_Ave_ = ∑*A*_*i*_τ_*i*_^2^/∑*A*_*i*_τ_*i*_,^40^ where *A*_*i*_ and τ_*i*_ represent the decay amplitude and component. The τ_Ave_ value of M_8 μg was higher (∼10.3 ns) compared to that of PEDOT:PSS (∼9.6 ns), while further increasing the content of Ti_3_C_2_T_*x*_ reduced τ_Ave_, recording ∼10.2 and ∼9.9 ns for M_16 μg and M_32 μg, respectively. The reduction of τ_Ave_ in M_32 μg could be attributed to the clustering of Ti_3_C_2_T_*x*_ that induced charge recombination centers, which could be detrimental to the photovoltaic performance of the PSCs. Similar observations could be made from the observation of Urbach energy extracted from visible-light absorption spectroscopy (Figure S13). The Urbach energy of the tin-based perovskite deposited on the HTLs decreased from ∼0.127 eV for PEDOT:PSS to ∼0.118 eV for Ti_3_C_2_T_*x*_, indicating the formation of PEA_0.2_FA_0.8_SnI_3_ with fewer trap states.

To study the photovoltaic performance of the HTLs, PSCs with a device structure of ITO/HTLs/PEA_0.2_FA_0.8_SnI_3_/ICBA/BCP/Ag (as shown in the inset of Figure 2c) were fabricated and characterized. The *J*–*V* curves for the champion devices are shown in Figure 2c, while the photovoltaic performance parameters averaged from 10 devices are summarized in Table 1. Improvement in the photovoltaic performance can be observed after the incorporation of MXene in PEDOT:PSS, where the device with M_8 μg as the HTL showed the best performance (PCE ∼8.3%, *J*_SC_ ∼21.8 mA cm^–2^, *V*_OC_ ∼0.65 V, and fill factor (FF) ∼0.59). The PCE reproducibility in M_8 μg also significantly improved, showing a standard deviation of 0.3%. In a separate experiment, the M_8 μg-based device also shows the best stabilized photovoltaic performance (Figure S14). Such an improvement in the photovoltaic performance can be attributed to the synergistic effect of improved perovskite film formation, enhanced carrier lifetime, and better aligned energy level arising from the inclusion of Ti_3_C_2_T_*x*_ in PEDOT:PSS. For stability analysis, the nonencapsulated PSCs employing different HTLs were stored in the dark under an inert N_2_ atmosphere for 450 h (following the ISOS-D protocol). Figure 2d shows that the device with M_16 μg as the HTL retained 89% of its PCE after 450 h of testing, followed by 84%, 72%, and 64% for M_8 μg, PEDOT, and M_32 μg, respectively. The *J*–*V* hysteresis for all samples increased with time, where M_16 μg recorded the lowest hysteresis index (HI), as shown in the inset of Figure 2d. The HI strongly depends on (i) the perovskite intrinsic properties as well as the properties of the charge-selective layers and (ii) external factors such as the measurement protocol and atmospheric conditions. Because the only parameter changing in all of these devices is the HTL and its interface with the perovskite, the changes in the HI over time suggested a strong dependence on the HTL properties and the related interfacial processes. Similar trends have been reported in the literature for lead-based PSCs.^41−43^ The performance deterioration in M_32 μg could be attributed to the accumulation of MXene within the PEDOT:PSS matrix, which induced the formation of a deep potential well that acts as a charge trap. Other than that, the higher surface roughness in PEDOT and M_32 μg could also lead to poorer surface wettability, leading to pinhole formation and an inferior photovoltaic performance.

**Table 1:** *J*_SC_, *V*_OC_, FF, and PCE for PSC Devices, Averaged from 10 Devices as well as the Champion Device

sample	direction	*J*_SC_ (mA cm^–2^)	*V*_OC_ (V)	FF (%)	PCE (%)
PEDOT	forward	20.7 ± 0.4	0.60 ± 0.06	51.8 ± 3.6	6.3 ± 0.6
	reverse	20.6 ± 0.5	0.61 ± 0.02	49.1 ± 2.2	6.1 ± 0.4
M_8 μg	forward	22.2 ± 0.6	0.64 ± 0.02	53.9 ± 3.4	7.6 ± 0.3
	reverse	21.8 ± 0.7	0.61 ± 0.02	49.7 ± 3.1	6.5 ± 0.3
M_16 μg	forward	22.1 ± 0.7	0.61 ± 0.05	55.3 ± 3.2	7.2 ± 0.4
	reverse	21.8 ± 0.8	0.54 ± 0.03	48.1 ± 3.4	5.6 ± 0.3
M_32 μg	forward	22.0 ± 0.9	0.62 ± 0.05	51.2 ± 6.2	6.3 ± 0.5
	reverse	21.5 ± 1.2	0.57 ± 0.06	46.1 ± 6.5	5.5 ± 0.3

The evolution of *J*_SC_, *V*_OC_, and FF throughout the stability measurement is shown in Figure S15. *J*_SC_ and FF remain considerably constant throughout the measurement, while *V*_OC_ shows an obvious reduction, indicating that the performance degradation originated from deteriorating *V*_OC_. The reduction of *V*_OC_ could be attributed to the increasing interfacial charge recombination due to hole accumulation at the interface.^44,45^ The accumulation of positive charges at the HTL/tin-based perovskite interface could induce the interfacial oxidation of PEA_0.2_FA_0.8_SnI_3_, leading to degradation of the device. Due to the improved energy alignment in M_8 μg and M_16 μg, coupled with enhanced charge extraction efficiency, the stability of the tin-based perovskite could be significantly improved, minimizing the accumulation of positive charges.

Overall, embedding PEDOT:PSS with a low concentration of Ti_3_C_2_T_*x*_ MXene could effectively tune the work function of the HTL, improving the energy alignment between the HTL and tin-based perovskite. The presence of Ti_3_C_2_T_*x*_ within PEDOT:PSS also improves the film quality of the tin-based perovskite, with enhanced homogeneity in the contact potential, which favors efficient charge transport/extraction. Cumulatively, these effects lead to a prolonged carrier lifetime and enhanced charge extraction. These improvements are directly reflected in the improved photovoltaic performance of the tin-based PSCs. Among the investigated concentrations, 8 μg mL^–1^ Ti_3_C_2_T_*x*_@PEDOT:PSS resulted in the best PCE of 8.3% (*J*_SC_ ∼21.8 mA cm^–2^, *V*_OC_ ∼0.65 V, and FF ∼59%), a 13.5% relative improvement compared to 7.3% for PEDOT (*J*_SC_ ∼ 20.4 mA cm^–2^, *V*_OC_ ∼ 0.60 V, and FF ∼59.9%). The device fabricated using Ti_3_C_2_T_*x*_-embedded PEDOT:PSS as the HTL also demonstrated improved stability, retaining 89% of its initial PCE after 450 h under an inert atmosphere, while its pristine PEDOT:PSS analogue retained only 72%.

## Supporting Information Available

Experimental details, schematics on the preparation of MXenes, details on the concentrated solution of the delaminated (single-layered) Ti_3_C_2_T_*x*_ MXenes, XRD, TEM, and XPS analysis of delaminated (single-layered) MXenes, work function analysis using PESA and KPFM, cross-section SEM, grain size distribution of tin-based perovskite films deposited on the HTLs, steady-state PL spectra for tin-based perovskite films deposited on the HTLs, Urbach energy of the tin-based perovskite film extracted from visible-light absorption spectroscopy, maximum power point tracking of devices, and photovoltaic parameter evolution during stability testing (PDF)

## References

[ref1] Li J.; Cao H.-L.; Jiao W.-B.; Wang Q.; Wei M.; Cantone I.; Lü J.; Abate A. Biological impact of lead from halide perovskites reveals the risk of introducing a safe threshold. Nat. Commun. 2020, 11 (1), 310 10.1038/s41467-019-13910-y. 31964862 PMC6974608

[ref2] Moody N.; Sesena S.; deQuilettes D. W.; Dou B. D.; Swartwout R.; Buchman J. T.; Johnson A.; Eze U.; Brenes R.; Johnston M.; Haynes C. L.; Bulović V.; Bawendi M. G. Assessing the Regulatory Requirements of Lead-Based Perovskite Photovoltaics. Joule 2020, 4 (5), 970–974. 10.1016/j.joule.2020.03.018.

[ref3] Wu P.; Zhang F. Recent Advances in Lead Chemisorption for Perovskite Solar Cells. Transactions of Tianjin University 2022, 28 (5), 341–357. 10.1007/s12209-022-00316-z.

[ref4] Jiang X.; Li H.; Zhou Q.; Wei Q.; Wei M.; Jiang L.; Wang Z.; Peng Z.; Wang F.; Zang Z.; Xu K.; Hou Y.; Teale S.; Zhou W.; Si R.; Gao X.; Sargent E. H.; Ning Z. One-Step Synthesis of SnI2·(DMSO)x Adducts for High-Performance Tin Perovskite Solar Cells. J. Am. Chem. Soc. 2021, 143 (29), 10970–10976. 10.1021/jacs.1c03032. 34196528

[ref5] Mahmoudi T.; Kohan M.; Rho W.-Y.; Wang Y.; Im Y. H.; Hahn Y.-B. Tin-Based Perovskite Solar Cells Reach Over 13% with Inclusion of N-Doped Graphene Oxide in Active, Hole-Transport, and Interfacial Layers. Adv. Energy Mater. 2022, 12 (43), 2201977 10.1002/aenm.202201977.

[ref6] Nishimura K.; Kamarudin M. A.; Hirotani D.; Hamada K.; Shen Q.; Iikubo S.; Minemoto T.; Yoshino K.; Hayase S. Lead-free tin-halide perovskite solar cells with 13% efficiency. Nano Energy 2020, 74, 104858 10.1016/j.nanoen.2020.104858.

[ref7] Yu B.-B.; Chen Z.; Zhu Y.; Wang Y.; Han B.; Chen G.; Zhang X.; Du Z.; He Z. Heterogeneous 2D/3D Tin-Halides Perovskite Solar Cells with Certified Conversion Efficiency Breaking 14%. Adv. Mater. 2021, 33 (36), 2102055 10.1002/adma.202102055. 34296476

[ref8] Zhu Z.; Jiang X.; Yu D.; Yu N.; Ning Z.; Mi Q. Smooth and Compact FASnI3 Films for Lead-Free Perovskite Solar Cells with over 14% Efficiency. ACS Energy Letters 2022, 7 (6), 2079–2083. 10.1021/acsenergylett.2c00776.

[ref9] Hou X.; Li F.; Zhang X.; Shi Y.; Du Y.; Gong J.; Xiao X.; Ren S.; Zhao X.-Z.; Tai Q. Reducing the Energy Loss to Achieve High Open-circuit Voltage and Efficiency by Coordinating Energy-Level Matching in Sn–Pb Binary Perovskite Solar Cells. Solar RRL 2021, 5 (8), 2100287 10.1002/solr.202100287.

[ref10] Zhang X.; Wang S.; Zhu W.; Cao Z.; Wang A.; Hao F. The Voltage Loss in Tin Halide Perovskite Solar Cells: Origins and Perspectives. Adv. Funct. Mater. 2022, 32 (8), 2108832 10.1002/adfm.202108832.

[ref11] Aldamasy M.; Iqbal Z.; Li G.; Pascual J.; Alharthi F.; Abate A.; Li M. Challenges in tin perovskite solar cells. Phys. Chem. Chem. Phys. 2021, 23 (41), 23413–23427. 10.1039/D1CP02596A. 34533139

[ref12] Ling J.; Kizhakkedath P. K. K.; Watson T. M.; Mora-Seró I.; Schmidt-Mende L.; Brown T. M.; Jose R. A Perspective on the Commercial Viability of Perovskite Solar Cells. Solar RRL 2021, 5 (11), 2100401 10.1002/solr.202100401.

[ref13] Liu J.; Ozaki M.; Yakumaru S.; Handa T.; Nishikubo R.; Kanemitsu Y.; Saeki A.; Murata Y.; Murdey R.; Wakamiya A. Lead-Free Solar Cells based on Tin Halide Perovskite Films with High Coverage and Improved Aggregation. Angew. Chem., Int. Ed. 2018, 57 (40), 13221–13225. 10.1002/anie.201808385. 30110137

[ref14] Pitaro M.; Tekelenburg E. K.; Shao S.; Loi M. A. Tin Halide Perovskites: From Fundamental Properties to Solar Cells. Adv. Mater. 2022, 34 (1), 2105844 10.1002/adma.202105844. PMC11469212 34626031

[ref15] Yao Y.; Cheng C.; Zhang C.; Hu H.; Wang K.; De Wolf S. Organic Hole-Transport Layers for Efficient, Stable, and Scalable Inverted Perovskite Solar Cells. Adv. Mater. 2022, 34 (44), 2203794 10.1002/adma.202203794. 35771986

[ref16] Yu J. C.; Hong J. A.; Jung E. D.; Kim D. B.; Baek S.-M.; Lee S.; Cho S.; Park S. S.; Choi K. J.; Song M. H. Highly efficient and stable inverted perovskite solar cell employing PEDOT:GO composite layer as a hole transport layer. Sci. Rep. 2018, 8 (1), 1070 10.1038/s41598-018-19612-7. 29348661 PMC5773582

[ref17] Fan X.; Nie W.; Tsai H.; Wang N.; Huang H.; Cheng Y.; Wen R.; Ma L.; Yan F.; Xia Y. PEDOT:PSS for Flexible and Stretchable Electronics: Modifications, Strategies, and Applications. Advanced Science 2019, 6 (19), 1900813 10.1002/advs.201900813. 31592415 PMC6774040

[ref18] Gebremichael Z. T.; Ugokwe C.; Alam S.; Stumpf S.; Diegel M.; Schubert U. S.; Hoppe H. How varying surface wettability of different PEDOT:PSS formulations and their mixtures affects perovskite crystallization and the efficiency of inverted perovskite solar cells. RSC Adv. 2022, 12 (39), 25593–25604. 10.1039/D2RA03843A. 36199329 PMC9453573

[ref19] Han W.; Ren G.; Liu J.; Li Z.; Bao H.; Liu C.; Guo W. Recent Progress of Inverted Perovskite Solar Cells with a Modified PEDOT:PSS Hole Transport Layer. ACS Appl. Mater. Interfaces 2020, 12 (44), 49297–49322. 10.1021/acsami.0c13576. 33089987

[ref20] Zhang R.; Ling H.; Lu X.; Xia J. The facile modification of PEDOT:PSS buffer layer by polyethyleneglycol and their effects on inverted perovskite solar cell. Sol. Energy 2019, 186, 398–403. 10.1016/j.solener.2019.05.018.

[ref21] Karimipour M.; Khazraei S.; Kim B. J.; Boschloo G.; Johansson E. M. J. Efficiency and Stability Enhancement of Perovskite Solar Cells Utilizing a Thiol Ligand and MoS2 (100) Nanosheet Surface Modification. ACS Applied Energy Materials 2021, 4 (12), 14080–14092. 10.1021/acsaem.1c02412.

[ref22] Karimipour M.; Paingott Parambil A.; Tabah Tanko K.; Zhang T.; Gao F.; Lira-Cantu M. Functionalized MXene/Halide Perovskite Heterojunctions for Perovskite Solar Cells Stable Under Real Outdoor Conditions. Adv. Energy Mater. 2023, 13 (44), 2301959 10.1002/aenm.202301959.

[ref23] Alhabeb M.; Maleski K.; Anasori B.; Lelyukh P.; Clark L.; Sin S.; Gogotsi Y. Guidelines for Synthesis and Processing of Two-Dimensional Titanium Carbide (Ti3C2Tx MXene). Chem. Mater. 2017, 29 (18), 7633–7644. 10.1021/acs.chemmater.7b02847.

[ref24] Din M. F. U.; Sousani S.; Kotlar M.; Ullah S.; Gregor M.; Scepka T.; Soyka Y.; Stepura A.; Shaji A.; Igbari F.; Vegso K.; Nadazdy V.; Siffalovic P.; Jergel M.; Omastova M.; Majkova E. Tailoring the electronic properties of the SnO2 nanoparticle layer for n-i-p perovskite solar cells by Ti3C2TX MXene. Materials Today Communications 2023, 36, 106700 10.1016/j.mtcomm.2023.106700.

[ref25] Urbančič J.; Tomsič E.; Chhikara M.; Pastukhova N.; Tkachuk V.; Dixon A.; Mavrič A.; Hashemi P.; Sabaghi D.; Nia A. S.; Bratina G.; Pavlica E. Time-of-flight photoconductivity investigation of high charge carrier mobility in Ti3C2Tx MXenes thin-film. Diamond Relat. Mater. 2023, 135, 109879 10.1016/j.diamond.2023.109879.

[ref26] Guo Z.; Gao L.; Xu Z.; Teo S.; Zhang C.; Kamata Y.; Hayase S.; Ma T. High Electrical Conductivity 2D MXene Serves as Additive of Perovskite for Efficient Solar Cells. Small 2018, 14 (47), 1802738 10.1002/smll.201802738. 30300503

[ref27] Yang Y.; Lu H.; Feng S.; Yang L.; Dong H.; Wang J.; Tian C.; Li L.; Lu H.; Jeong J.; Zakeeruddin S. M.; Liu Y.; Grätzel M.; Hagfeldt A. Modulation of perovskite crystallization processes towards highly efficient and stable perovskite solar cells with MXene quantum dot-modified SnO2. Energy Environ. Sci. 2021, 14 (6), 3447–3454. 10.1039/D1EE00056J.

[ref28] Saranin D.; Pescetelli S.; Pazniak A.; Rossi D.; Liedl A.; Yakusheva A.; Luchnikov L.; Podgorny D.; Gostischev P.; Didenko S.; Tameev A.; Lizzit D.; Angelucci M.; Cimino R.; Larciprete R.; Agresti A.; Di Carlo A. Transition metal carbides (MXenes) for efficient NiO-based inverted perovskite solar cells. Nano Energy 2021, 82, 105771 10.1016/j.nanoen.2021.105771.

[ref29] Chen X.; Xu W.; Ding N.; Ji Y.; Pan G.; Zhu J.; Zhou D.; Wu Y.; Chen C.; Song H. Dual Interfacial Modification Engineering with 2D MXene Quantum Dots and Copper Sulphide Nanocrystals Enabled High-Performance Perovskite Solar Cells. Adv. Funct. Mater. 2020, 30 (30), 2003295 10.1002/adfm.202003295.

[ref30] Chava V. S. N.; Chandrasekhar P. S.; Gomez A.; Echegoyen L.; Sreenivasan S. T. MXene-Based Tailoring of Carrier Dynamics, Defect Passivation, and Interfacial Band Alignment for Efficient Planar p–i–n Perovskite Solar Cells. ACS Applied Energy Materials 2021, 4 (11), 12137–12148. 10.1021/acsaem.1c01669.

[ref31] Liu Y.; Tao Q.; Jin Y.; Liu X.; Sun H.; Ghazaly A. E.; Fabiano S.; Li Z.; Luo J.; Rosen J.; Zhang F. Mo1.33C MXene-Assisted PEDOT:PSS Hole Transport Layer for High-Performance Bulk-Heterojunction Polymer Solar Cells. ACS Applied Electronic Materials 2020, 2 (1), 163–169. 10.1021/acsaelm.9b00668.

[ref32] Zheng Z.; Hu Q.; Zhang S.; Zhang D.; Wang J.; Xie S.; Wang R.; Qin Y.; Li W.; Hong L.; Liang N.; Liu F.; Zhang Y.; Wei Z.; Tang Z.; Russell T. P.; Hou J.; Zhou H. A Highly Efficient Non-Fullerene Organic Solar Cell with a Fill Factor over 0.80 Enabled by a Fine-Tuned Hole-Transporting Layer. Adv. Mater. 2018, 30 (34), 1801801 10.1002/adma.201801801. 29989212

[ref33] Hussain S.; Liu H.; Hussain M.; Mehran M. T.; Kim H.-S.; Jung J.; Vikraman D.; Kang J. Development of MXene/WO3 embedded PEDOT:PSS hole transport layers for highly efficient perovskite solar cells and X-ray detectors. International Journal of Energy Research 2022, 46 (9), 12485–12497. 10.1002/er.8020.

[ref34] Chen X.; Liu Y.; Sun Y.; Zhao T.; Zhao C.; Khattab T. A.; Lim E. G.; Sun X.; Wen Z. Electron trapping & blocking effect enabled by MXene/TiO2 intermediate layer for charge regulation of triboelectric nanogenerators. Nano Energy 2022, 98, 107236 10.1016/j.nanoen.2022.107236.

[ref35] Ovodok E. A.; Ivanovskaya M. I.; Poznyak S. K.; Maltanova A. M.; Azarko I. I.; Micusik M.; Omastava M.; Aniskevich A. Synthesis of Ti3AlC2 max phase under vacuum, its structural characterization and using for Ti3C2Tx MXene preparation. Thin Solid Films 2023, 771, 139759 10.1016/j.tsf.2023.139759.

[ref36] Caffrey N. M. Effect of mixed surface terminations on the structural and electrochemical properties of two-dimensional Ti3C2T2 and V2CT2MXenes multilayers. Nanoscale 2018, 10 (28), 13520–13530. 10.1039/C8NR03221A. 29972202

[ref37] Chen H.; Maxwell A.; Li C.; Teale S.; Chen B.; Zhu T.; Ugur E.; Harrison G.; Grater L.; Wang J.; Wang Z.; Zeng L.; Park S. M.; Chen L.; Serles P.; Awni R. A.; Subedi B.; Zheng X.; Xiao C.; Podraza N. J.; Filleter T.; Liu C.; Yang Y.; Luther J. M.; De Wolf S.; Kanatzidis M. G.; Yan Y.; Sargent E. H. Regulating surface potential maximizes voltage in all-perovskite tandems. Nature 2023, 613 (7945), 676–681. 10.1038/s41586-022-05541-z. 36379225

[ref38] Cuzzupè D. T.; Öz S. D.; Ling J.; Illing E.; Seewald T.; Jose R.; Olthof S.; Fakharuddin A.; Schmidt-Mende L., Understanding the Methylammonium Chloride-Assisted Crystallization for Improved Performance of Lead–Free Tin Perovskite Solar Cells. Solar RRL 2023, 7 ( (24), ). 10.1002/solr.202300770.

[ref39] Péan E. V.; Dimitrov S.; De Castro C. S.; Davies M. L. Interpreting time-resolved photoluminescence of perovskite materials. Phys. Chem. Chem. Phys. 2020, 22 (48), 28345–28358. 10.1039/D0CP04950F. 33300902

[ref40] Jegorovė A.; Xia J.; Steponaitis M.; Daskeviciene M.; Jankauskas V.; Gruodis A.; Kamarauskas E.; Malinauskas T.; Rakstys K.; Alamry K. A.; Getautis V.; Nazeeruddin M. K. Branched Fluorenylidene Derivatives with Low Ionization Potentials as Hole-Transporting Materials for Perovskite Solar Cells. Chem. Mater. 2023, 35 (15), 5914–5923. 10.1021/acs.chemmater.3c00708. 37576588 PMC10413965

[ref41] Habisreutinger S. N.; Noel N. K.; Snaith H. J. Hysteresis Index: A Figure without Merit for Quantifying Hysteresis in Perovskite Solar Cells. ACS Energy Letters 2018, 3 (10), 2472–2476. 10.1021/acsenergylett.8b01627.

[ref42] Haider M. I.; Hu H.; Seewald T.; Ahmed S.; Sultan M.; Schmidt-Mende L.; Fakharuddin A. Ethylenediamine Vapors-Assisted Surface Passivation of Perovskite Films for Efficient Inverted Solar Cells. Solar RRL 2023, 7 (9), 2201092 10.1002/solr.202201092.

[ref43] Tress W. Metal Halide Perovskites as Mixed Electronic–Ionic Conductors: Challenges and Opportunities—From Hysteresis to Memristivity. J. Phys. Chem. Lett. 2017, 8 (13), 3106–3114. 10.1021/acs.jpclett.7b00975. 28641009

[ref44] Daboczi M.; Hamilton I.; Xu S.; Luke J.; Limbu S.; Lee J.; McLachlan M. A.; Lee K.; Durrant J. R.; Baikie I. D.; Kim J.-S. Origin of Open-Circuit Voltage Losses in Perovskite Solar Cells Investigated by Surface Photovoltage Measurement. ACS Appl. Mater. Interfaces 2019, 11 (50), 46808–46817. 10.1021/acsami.9b16394. 31738042

[ref45] Rutledge S. A.; Helmy A. S. Carrier mobility enhancement in poly(3,4-ethylenedioxythiophene)-poly(styrenesulfonate) having undergone rapid thermal annealing. J. Appl. Phys. 2013, 114 (13), 133708 10.1063/1.4824104.

