# Supplementary material for: MXene-Embedded PEDOT:PSS Hole-Transport Material for Lead-Free Perovskite Solar Cells
Source: ACS Appl Energy Mater. 2024 Aug 23;7(17):7152–8. doi: 10.1021/acsaem.3c02928 (PMC11389724; doi:10.1021/acsaem.3c02928)
Supplement: Supplementary file 1 — ae3c02928_si_001.pdf [file ae3c02928_si_001.pdf]

Supporting Information

MXene-Embedded PEDOT:PSS Hole Transport Material for Lead-Free Perovskite Solar Cells

JinKiong Ling[‡], Daniele T. Cuzzupè[‡], Muhammad Faraz Ud Din, Anastasiia Stepura, Tom Burgard, Yekitwork Abebe Temitmie, Eva Majkova, Maria Omastova, Rajan Jose, Lukas Schmidt-Mende*, and Azhar Fakharuddin*

[‡]*These authors contributed equally.*

**Corresponding author: L. Schmidt-Mende (Lukas.Schmidt-Mende@uni-konstanz.de); A. Fakharuddin (azhar-fakhar.uddin@uni-konstanz.de)*

Contents:

Number of pages: 17

Number of tables: 1

Number of figures: 15

EXPERIMENTAL SECTIONS

Materials and Methods

ITO substrates were acquired from *Luminescence Technology Corp* (thickness 2 mm, ~15 Ω/□), formamidinium iodide (>99%) and PEAI (>98%) from *Greatcell Solar*, dimethylformamide (99.8%, extra dry) and dimethyl sulfoxide (99.7+%, extra dry) were purchased from *Acros Organics*, SnI_2 (>97.0%), chlorobenzene (anhydrous, 99.8%) and SnF_2 (99%) from *Sigma Aldrich*, ICBA (>98.5%) and BCP (>99.0%) were acquired from *Ossila*. These chemicals were used without further purification or processing. PEDOT:PSS (Clevios PV P AI 4083 dispersion in water) was obtained from *Heraeus*. The PEDOT:PSS solution was filtered through a 0.45 μm PVDF filter prior to usage.

Synthesis of $Ti_3C_2T_x$ MXene

For etching of Ti_3AlC_2 MAX phase, 9M HCl was poured into Teflon bottle immersed into cold water bath. Afterwards, during continuous stirring, 5 g of LiF were mixed to the HCl and further mixed for 5 minutes. Subsequently, 5 g of Ti_3AlC_2 MAX phase were slowly added to the mixture. After stirring for 24 hours at room temperature, the mixture was washed with deionized water through centrifugation cycles until the pH value of the solution reaches above 6. The obtained paste-like product (containing multi-layered MXene) was collected into a glass bottle and stored in the fridge. To obtain delaminated single-layered MXenes solution, the MXenes paste was mixed with LiCl (ratio — 1 g of $Ti_3C_2T_x$: 1 g of LiCl) in 20 mL of deionized water. The mixture was magnetically stirred for 24 hours in 35°C water bath. The reacted mixture was centrifuged repetitively at 3500 rpm for 10 minutes until the supernatant turned black. Afterwards, it was centrifuged for 1 hour for few cycles until the supernatant turned dark green, an indication that the supernatant contains delaminated MXene sheets. The supernatant was collected and the centrifugation-collection process was repeated until the supernatant

became slightly green or transparent. The concentration of the prepared solution was calculated by vacuum-assisted filtering 20 mL of the collected supernatant using Celgard®3501 membrane, resulting in a thin MXene film on the filter membrane. The MXene film was vacuum dried at 45°C overnight, then it was weighed on a balance. The concentration of the MXene solution was measured to be 0.64 mg/mL. To further increase the concentration of the MXene, the collected solution was evaporated using rotary vacuum evaporator until a final solution concentration of 6.4 mg/mL.

MXenes Characterization

Surface properties of MAX phase and delaminated MXenes were studied with X-ray Photoelectron Spectroscopy (XPS). XPS signals were recorded using a Thermo Scientific K-Alpha XPS system (Thermo Fisher Scientific, UK) equipped with a micro-focused, monochromatic Al Kα X-ray source (1486.67 eV). An X-ray beam of 400 μm size was used at 6 mA and 12 kV. The spectra were acquired in the constant analyzer energy mode with pass energy of 200 eV for the survey. Narrow regions were collected using the pass energy of 50 eV. Charge compensation was achieved with the system dual beam flood gun. The Thermo Scientific Avantage software, version 5.9911 (Thermo Fisher Scientific), was used for digital acquisition and data processing. Spectral calibration was determined by using the automated calibration routine and the internal Au, Ag, and Cu standards supplied with the K-Alpha system. The surface compositions (in atomic %) were determined by considering the integrated peak areas of the detected atoms and the respective sensitivity factors. The crystal structure of the MXenes was analysed using Philips X-ray PANalytical Empyrean diffractometer with Cu Kα radiation (λ = 1.5406 Å) for 2θ ranging 2-70°. LEO-906E transmission electron microscope (operating voltage 100 kV) was used to analyse the morphological features of the samples.

Device Fabrication

The ITO sheets were cut into 14 × 14 mm^2 substrates, followed by cleaning using ultrasonication for 30 minutes in the sequence of detergent (2% Hellmanex III solution), deionized water, acetone, and iso-propanol. The washed ITO substrates were dried and treated with UV/Ozone treatment for 30 minutes. The MXene@PEDOT:PSS solution was prepared by dispersing 2, 4, and 8 µL of 6.4 mg/mL MXene solution in 200 µL of PEDOT:PSS solution through ultrasonication for 2 minutes. The resultant MXene concentrations in the mixtures were 8, 16, and 32 µg/mL, denoted as M_8µg, M_16µg, and M_32µg. The PEDOT:PSS and mixture embedded with MXene were spin-coated onto the pre-cleaned ITO substrates at 6000 rpm for 60 seconds, followed by annealing at 150°C for 20 minutes. The spin-coating was performed under a humidity-controlled chamber (humidity level between 20-30%). The $PEA_{0.2}FA_{0.8}SnI_3$ lead-free perovskite (LFP) precursor was prepared by dispersing 12.58 mg SnF_2, 298.02 mg SnI_2, 39.85 mg PEAI, and 110.06 mg FAI in 1 mL of DMF/DMSO solvent in a volume ratio of 4:1. The LFP solution with a concentration of 0.8 M (respect to the Sn content) was stirred overnight at room temperature and filtered through a 0.22 µm PTFE filter. In a nitrogen-filled glovebox with O_2 and H_2O content < 5 ppm, the resultant mixture was spin-coated onto the HTL-coated substrates at 8000 rpm for 60 seconds. Antisolvent treatment was performed 6 seconds before the end of the program through dropping 200 µL of chlorobenzene (pre-heated to 70°C). The coating was subsequently annealed at 80°C for 10 minutes. For the deposition of electron transport layers, ICBA in chlorobenzene (20 mg/mL) solution was spin-coated at 1200 rpm for 30 seconds followed by annealing at 70°C for 10 minutes. Subsequently, 0.5 mg/mL BCP in isopropanol solution was dynamically spin-coated at 5000 rpm for 30 seconds and annealed at 70°C for a minute. The device was completed by evaporating 100 nm of silver as the counter electrode. The evaporation was performed under a vacuum of $<4\times10^{-6}$ mbar at deposition rates of 0.2, 0.6, and 1.0 Å/s, to a film thickness of 10, 25, and 100 nm.

Device Characterization

Current density-voltage characteristics curves of the PSCs were recorded under equivalent 1 sun illumination with a step size of 0.05 V and a delay of 0.01 s in a nitrogen filled glovebox with an assembly of a Keithley 2400 SMU, and LOT 300W Xenon solar simulator calibrated with a Fraunhofer ISE-certified Si reference diode equipped with a Schott KG-5 filter. Maximum power point (MPP) tracking algorithm, developed by Zimmermann et al.[43], was employed for a reliable PSCs measurement. The stability of the fabricated devices was tested over 450 hours period while stored unencapsulated in inert N_2 atmosphere, following the ISOS-D protocol. Maximum power point tracking was performed under a constant 1 sun illumination for 10 minutes, with respective JV taken at 0.05 V and a delay of 0.01 s in a nitrogen-filled glovebox. Both steady-state photoluminescence (PL) and time-resolved photoluminescence (TRPL) were measured using *PicoQuant Fluo Time300* fluorescence spectrometer using a 405 nm excitation laser. The repetition rate was set to 40 MHz for steady state and 1 MHz for time-resolved measurements, respectively. To block stray laser light, a 455 nm long pass filter was placed between the sample and the detector. For long-term illumination, the steady state settings were used. UV-vis absorbance spectroscopy was performed with a *Cary 5000* UV/Vis spectrometer by *Agilent Technologies*. Atomic Force Microscopy of perovskite films was performed using a NX10 system by *Park Systems* in controlled humidity (below 3%). The humidity was controlled by flushing N_2 in the acoustic chamber. The KPFM images were recorded in side-band modulation mode for a better local resolution of the CPD value and using the ASYELEC.01-R2 tip without applying any sample bias. The KPFM measurements were recorded in the dark. All devices were unencapsulated, stored in ambient room light in nitrogen environment. And measured periodically to track their IV performance over time.

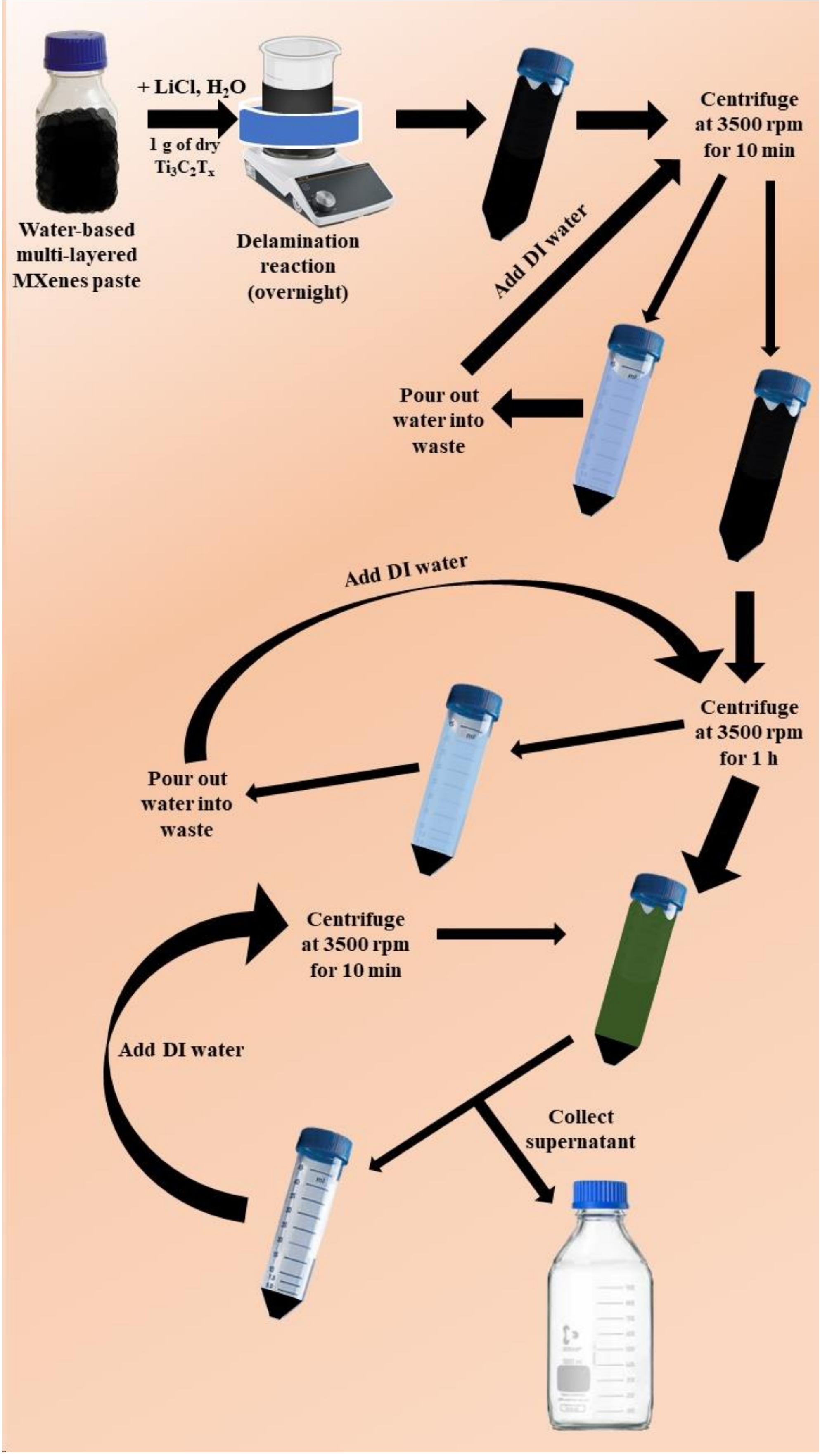

Figure S1. Schematic for obtaining delaminated (single-layered) $Ti_3C_2T_x$ MXenes.

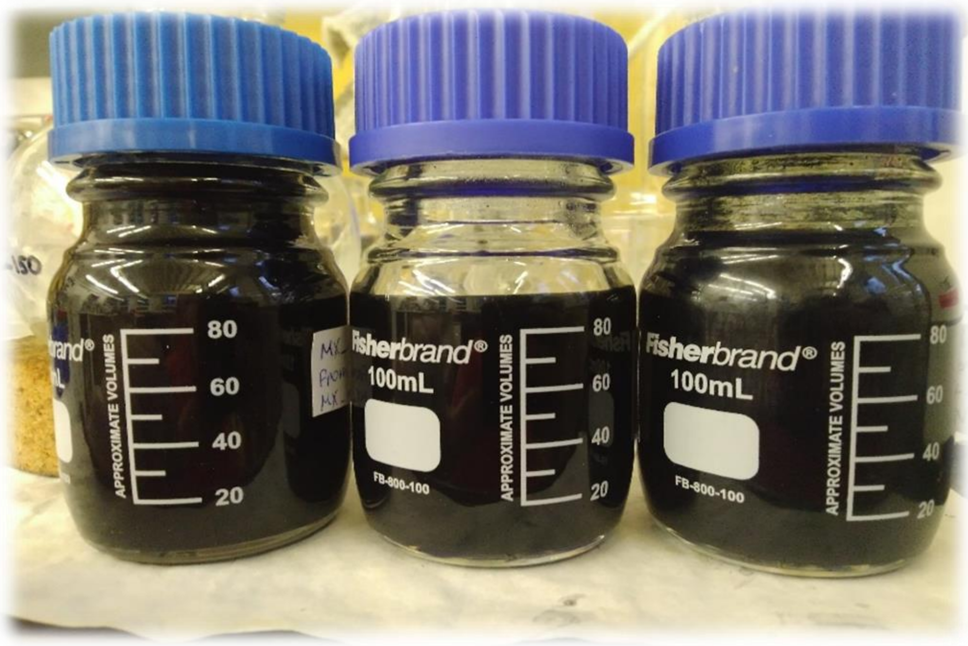

Figure S2. Concentrated solution of the delaminated (single-layered) $Ti_3C_2T_x$ MXenes.

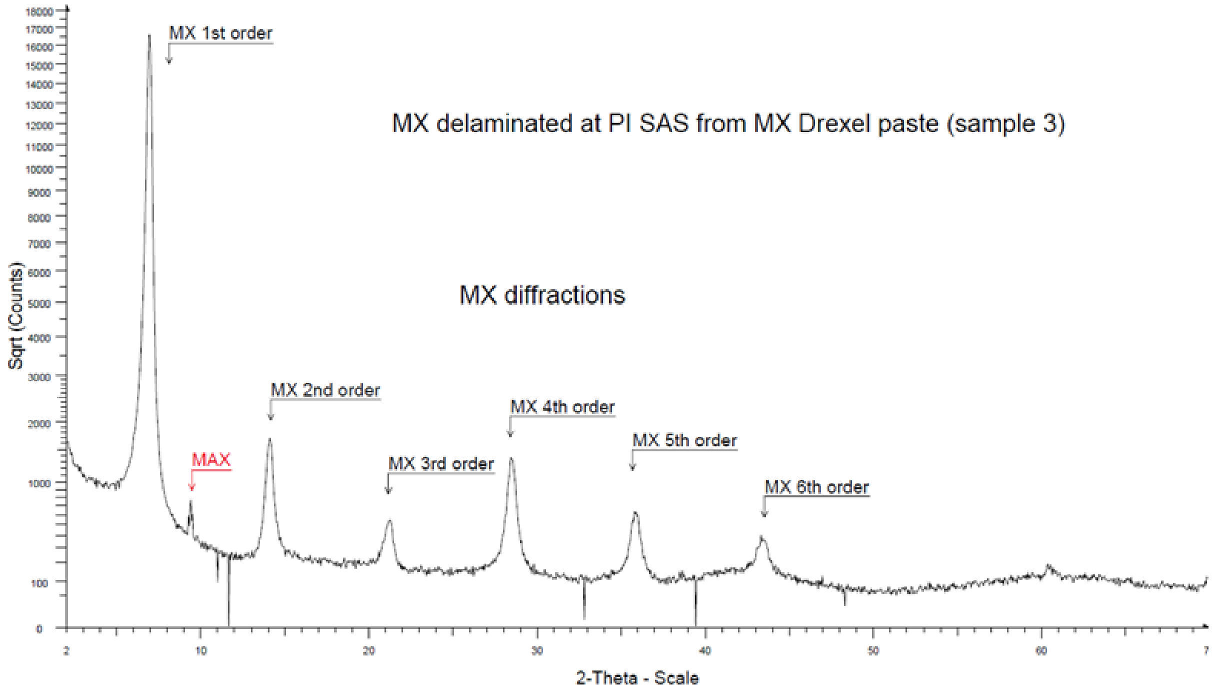

Figure S3. The XRD spectrum for the delaminated (single-layered) $Ti_3C_2T_x$ MXenes. Besides from the small diffraction peaks of MAX at 2θ ~10º (small amount of unreacted MAX phase), the XRD concluded that the resulting sample has high purity of delaminated MXenes phase.

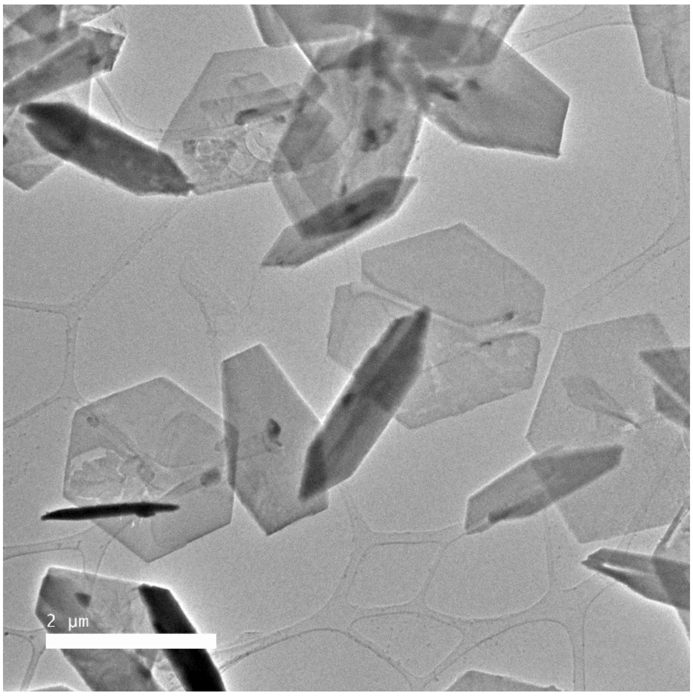

Figure S4. TEM images of the delaminated $Ti_3C_2T_x$ MXenes. The high transparency coupled with the thin edge concluded that the delaminated $Ti_3C_2T_x$ MXenes are of single layer.

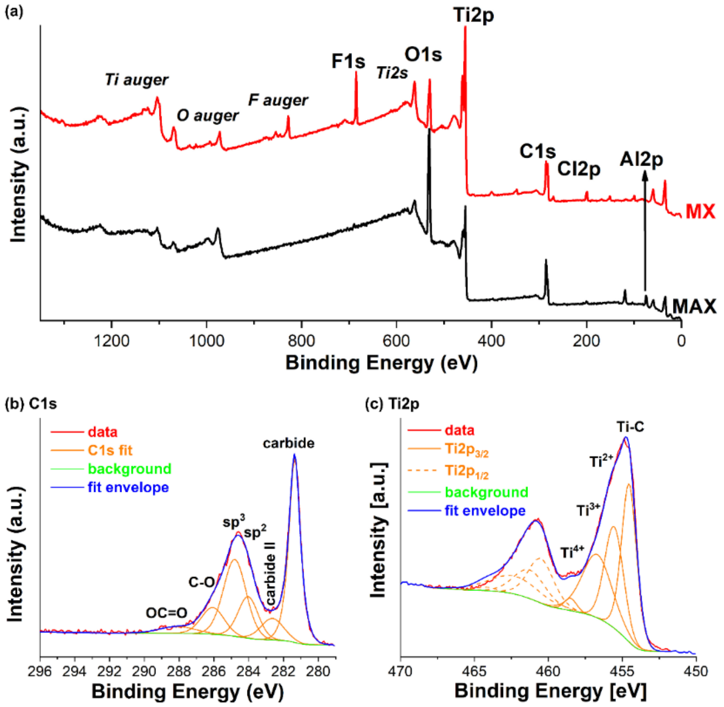

Figure S5. (a) The XPS survey scan of MAX phase and the prepared MXene. The XPS spectrum (technique focusing on the surface chemical state of the sample) of MXene shows the presence of -F and -O functional groups, where the absence of Al2p signal after exfoliation indicates the success of MXene synthesis. The high-resolution scan focusing on (b) C1s and (c) Ti2p states were also provided. A detailed analysis on the atomic% of each chemical state were provided in Table S1.

Table S1. Surface chemical composition of the MAX phase and MXene samples as determined by XPS.

Samples	Surface chemical composition [at.%]				
	C1s<br>Ti_3C_2/I/sp^2/sp^3/CO/OCO	O1s<br>ox/I/II/III	Ti2p<br>Ti_3C_2/I/II/TiO_2	F1s<br>F^-/XF/XFX/C-F	Cl2p/N1s/Al2p/Si2p
MAX phase	34.2<br>9.3/1.4/1.9/16.2/3.1/2.3	37.4<br>13.0/10.4/12.7/1.3	16.5<br>5.6/5.2/2.3/3.4	—	0.6/—/11.4/—
2D MXene	36.7<br>13.6/2.9/4.9/10.3/3.6/1.4	18.7<br>7.6/4.5/5.5/1.1	26.2<br>9.9/7.8/7.9/0.6	10.0<br>0.1/8.2/1.2/0.5	1.9/2.4/—/4.2

C1s: I – carbide II (or carbide asymmetry)

O1s: I – C=O, II – C-O, III - OCO

Ti2p: I – Ti^{2+}(or carbide asymmetry), II – Ti^{3+}

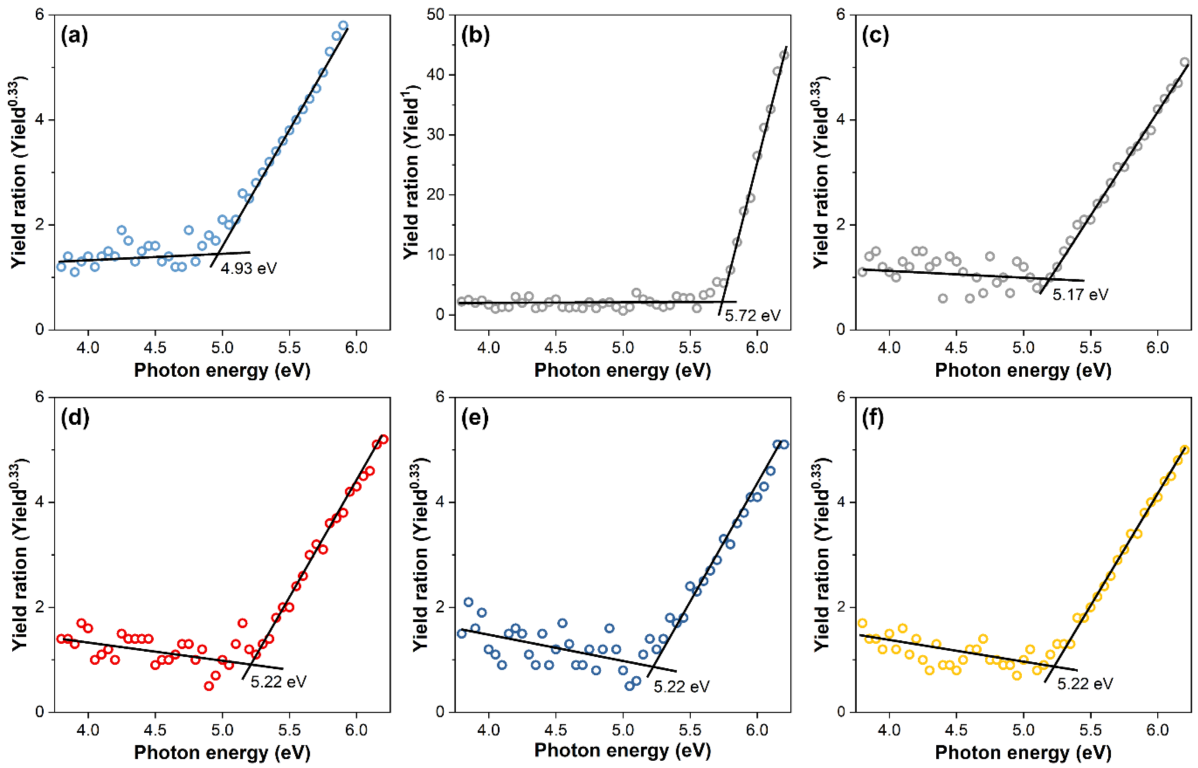

Figure S6. The PESA spectra for (a) bare ITO substrate, (b) MXene coating on ITO substrate, (c) pristine MXene, (d) 8 μg/mL Ti_3C_2@PEDOT, (e) 16 μg/mL Ti_3C_2@PEDOT, and (f) 32 μg/mL Ti_3C_2@PEDOT. All spectra (excluding that for bare ITO substrate) were analyzed at 10 nW power, while spectrum (a) was recorded at a power of 1000 nW. To prevent the impact of degraded MXene on the PESA analysis, the measurement was done immediately after each coated substrate was removed from the glovebox.

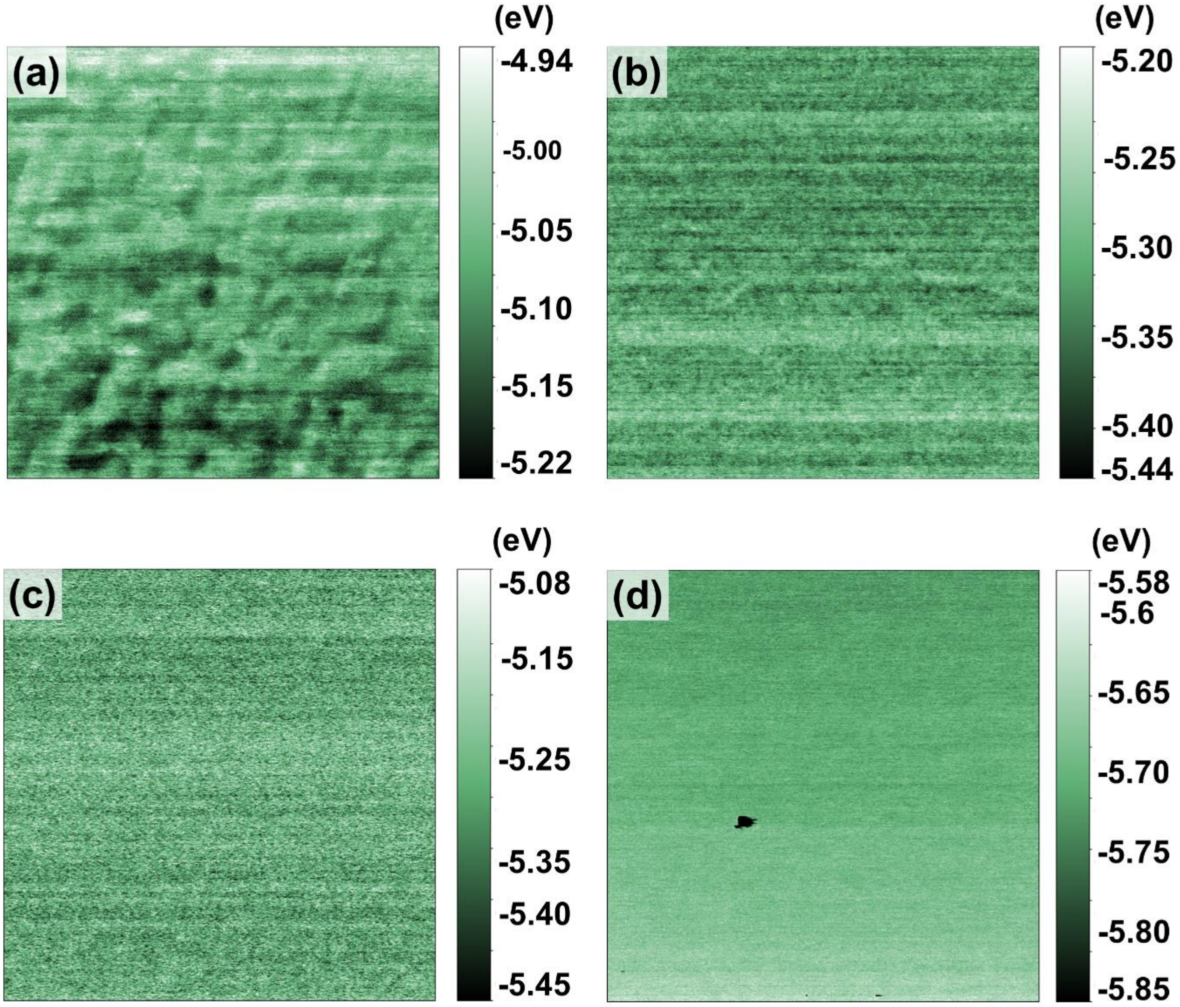

Figure S7. The local electrical properties of (a) pristine PEDOT, (b) M_8μg, (c) M_16μg, and (d) M_32μ films, respectively. The images show a scan area of 2×2 μm^2.

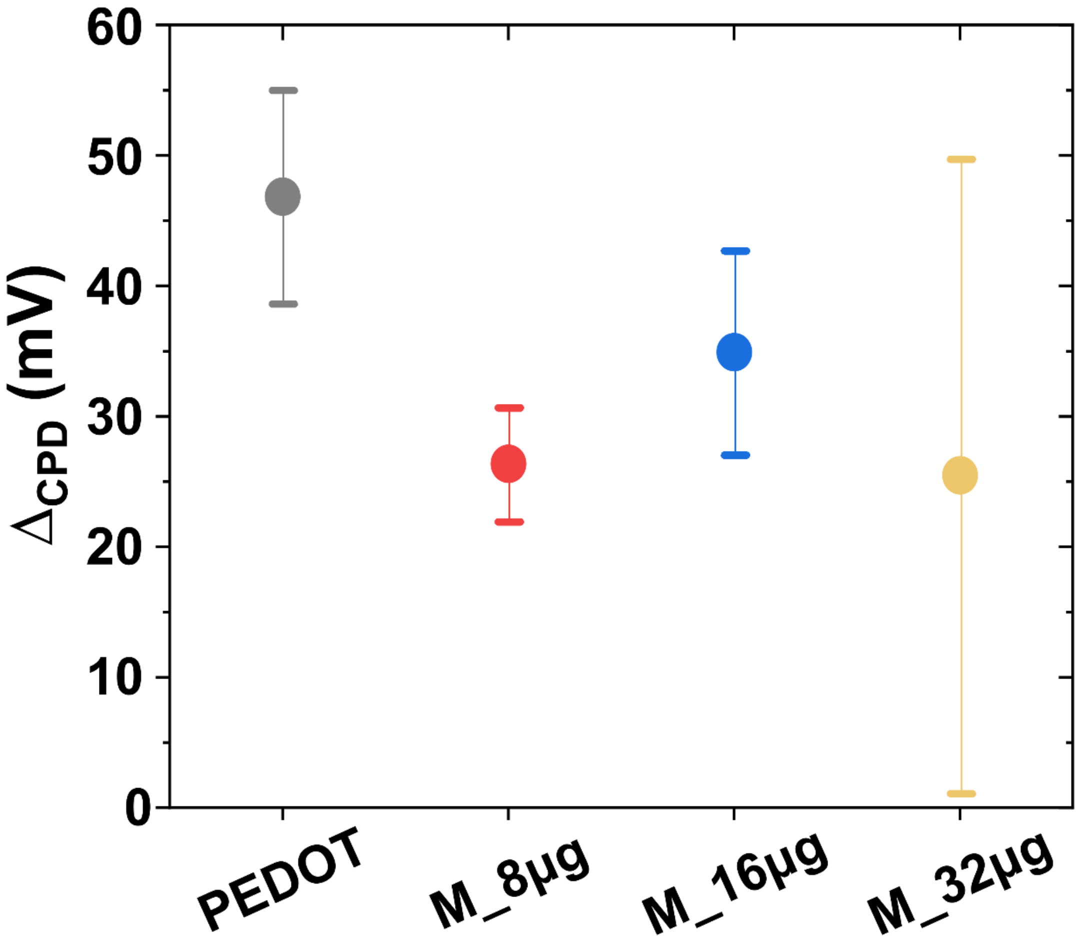

Figure S8. Statistical distribution (measured over at least 4 spots on two different samples for each type) of local variation in the contact potential difference (Δ_{CPD}) of all samples.

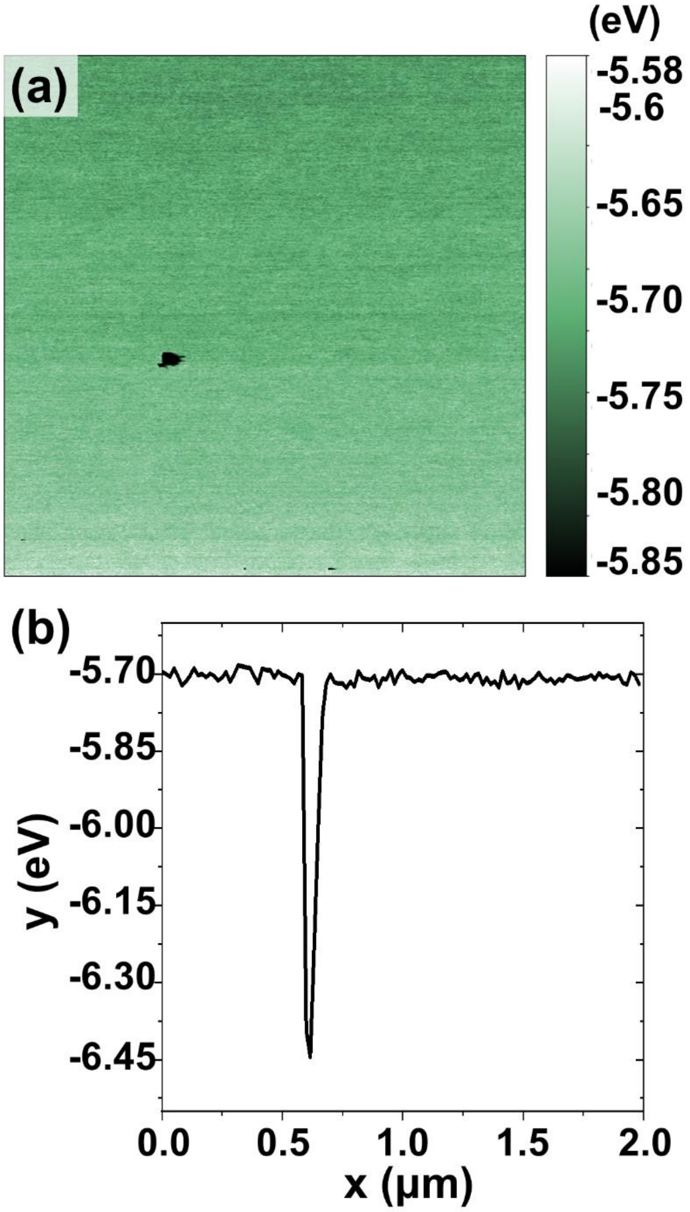

Figure S9. (a) The local electrical properties of M_32μ films and (b) the line profile over (a) to depict the local variation in Δ_{CPD}.

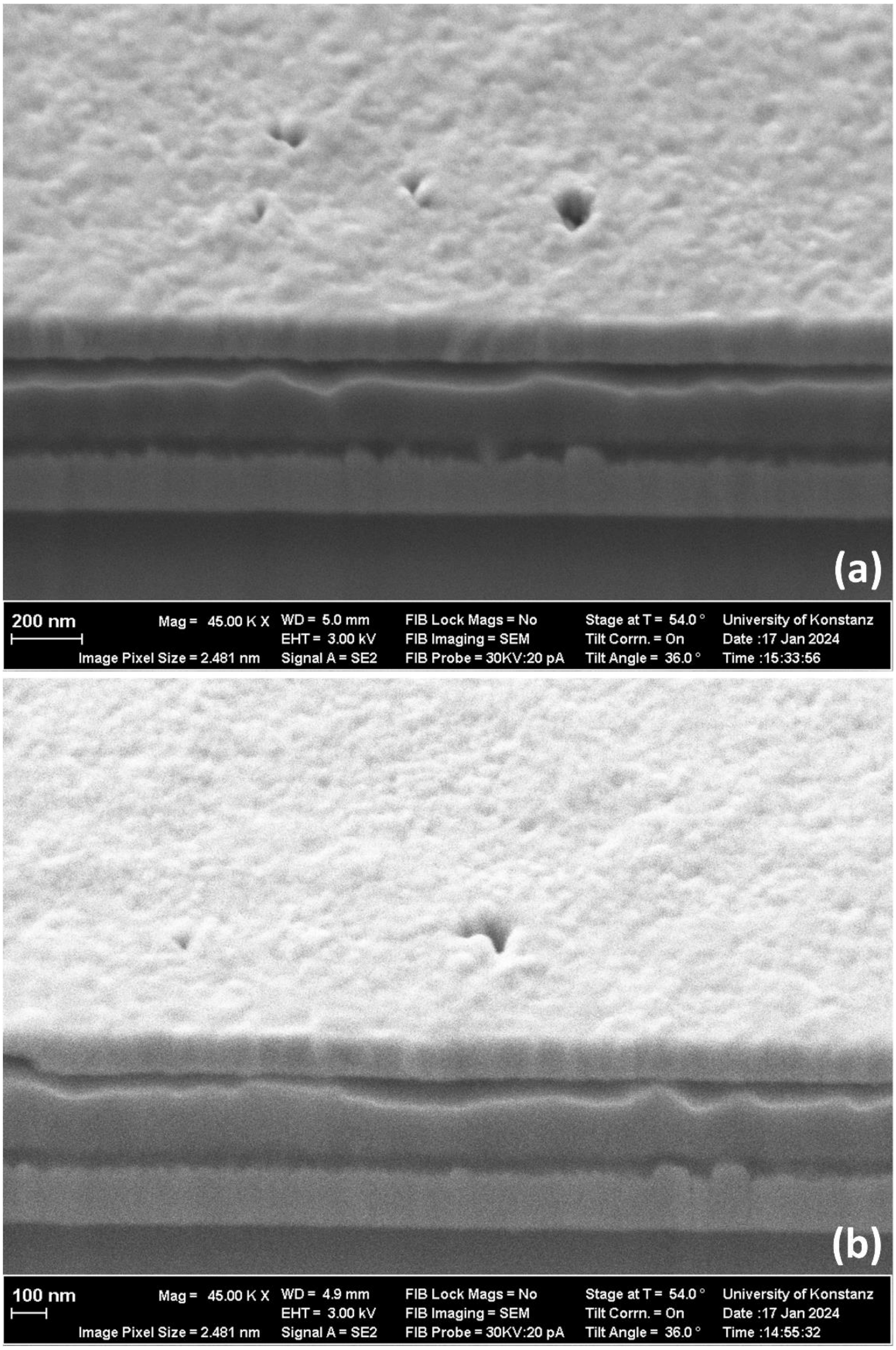

Figure S10. Cross-section FIB-cut SEM of the incomplete device with an architecture of glass/ITO/HTL/$PEA_{0.2}FA_{0.8}SnI_3$. Analyses were performed on the (a) pristine PEDOT:PSS HTL and (b) M_8μg HTL for comparison.

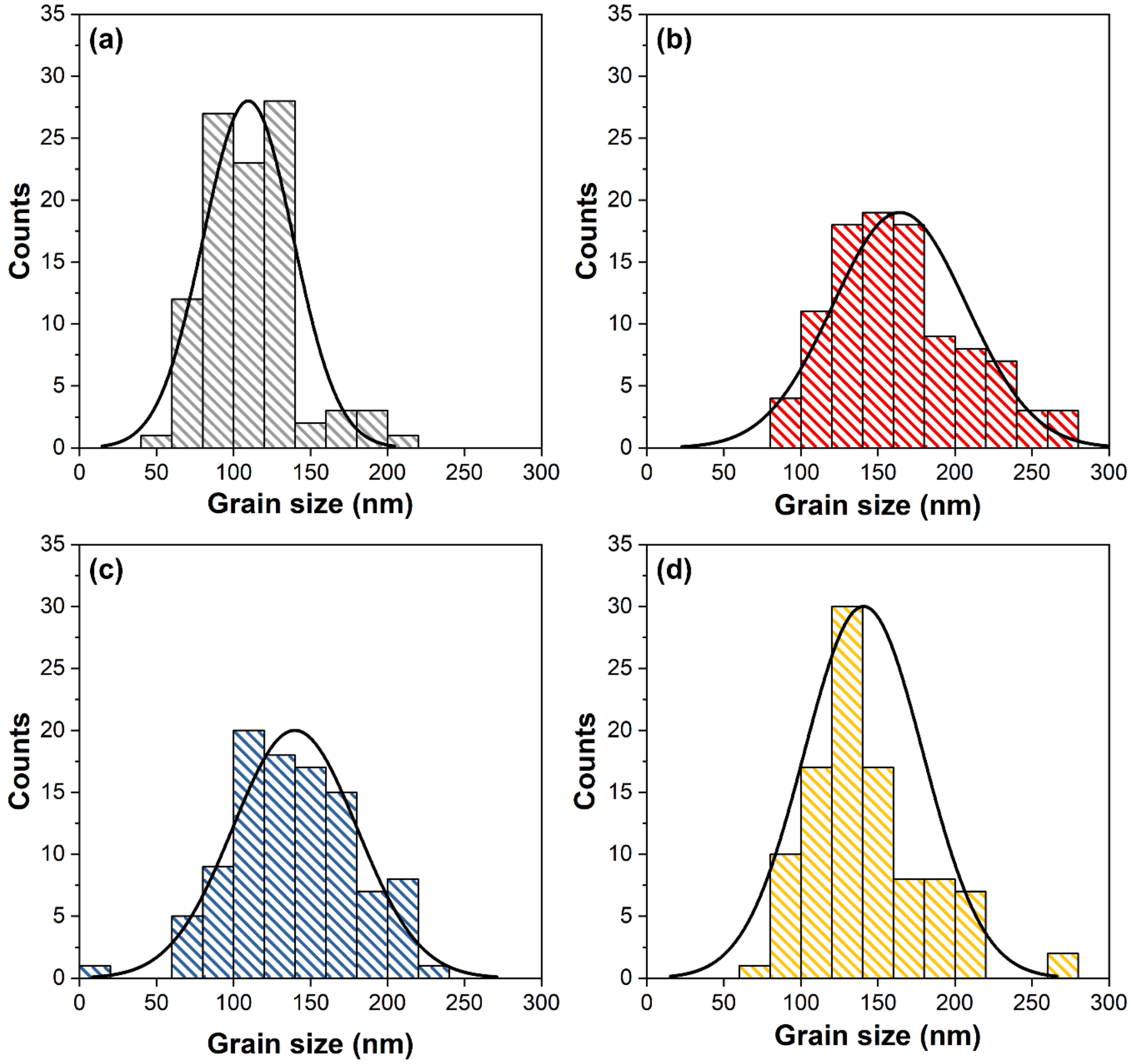

Figure S11. Distribution of grain size of tin-based perovskite deposited on (a) pristine PEDOT:PSS, (b) M_8μg, (c) M_16μg, and (d) M_32μg, respectively. The distribution was collected by measuring the width of 100 grains throughout the SEM images.

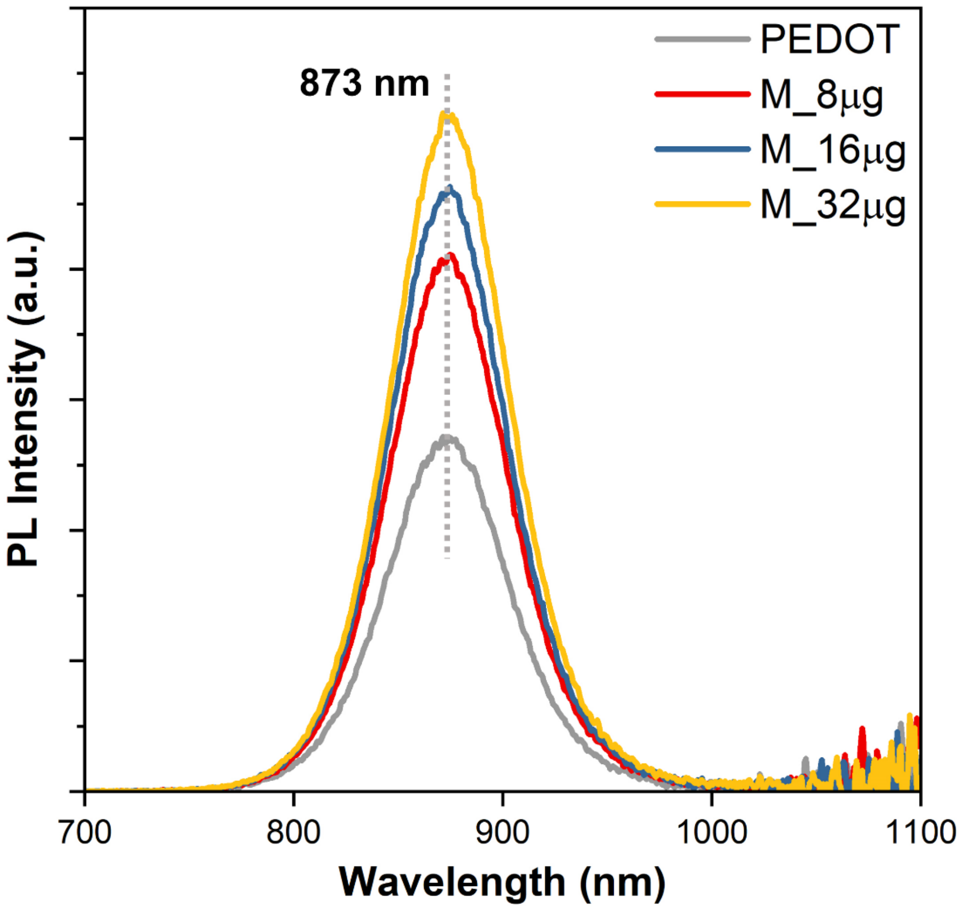

Figure S12. The steady-state photoluminescence spectra for all samples. The emission peaks for all samples centered at ~874 nm, equivalent to an optical energy gap of 1.42 eV.

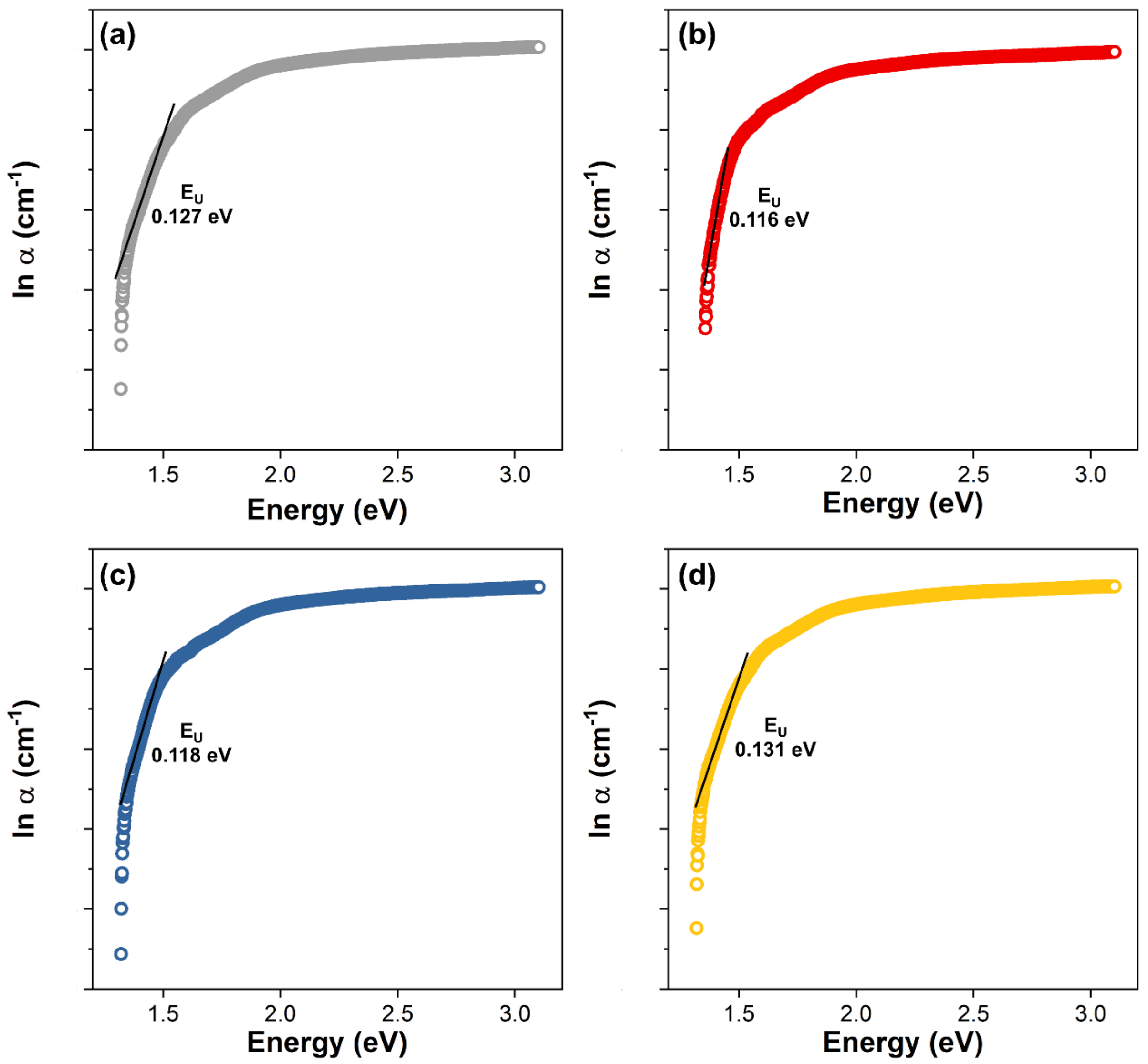

Figure S13. The Urbach energy (E_U) for (a) pristine PEDOT:PSS, (b) 8μg/mL Ti_3C_2@PEDOT, (c) 16 μg/mL Ti_3C_2@PEDOT, and (d) 32 μg/mL Ti_3C_2@PEDOT. The Urbach energy was extracted from the slope of the curve right after the optical energy gap, which would be 1.42 eV as calculated from the PL (Figure 2b). The higher Urbach energy in pristine PEDOT:PSS can be translated as wider energy trap states below (above) the conduction (valence) bands. Smaller Urbach energy is favourable for optoelectronic applications.

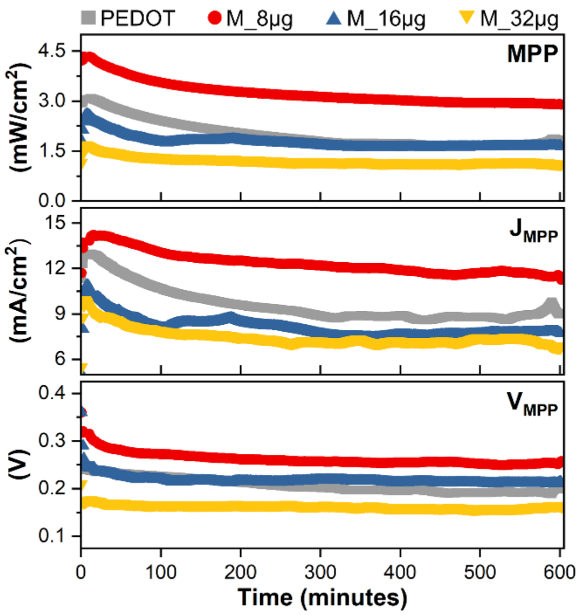

Figure S14. The maximum power point (MPP), photocurrent at MPP (J_{MPP}), and photovoltage at MPP (V_{MPP}) of the devices.

The experiment on maximum power tracking (MPP) was performed during the revision of the manuscript. Due to a 20 times higher O_2 and a 5 times higher H_2O concentration in the glovebox, the overall PCE of the device was lower than in the original experiments. This can be seen from the overall lower PCE of the device than those reported in the original work. However, the relative trend in the performance and the reproducibility of MXene inclusion and its beneficial impact on the device performance can be shown, which is in line with our previous experiments.

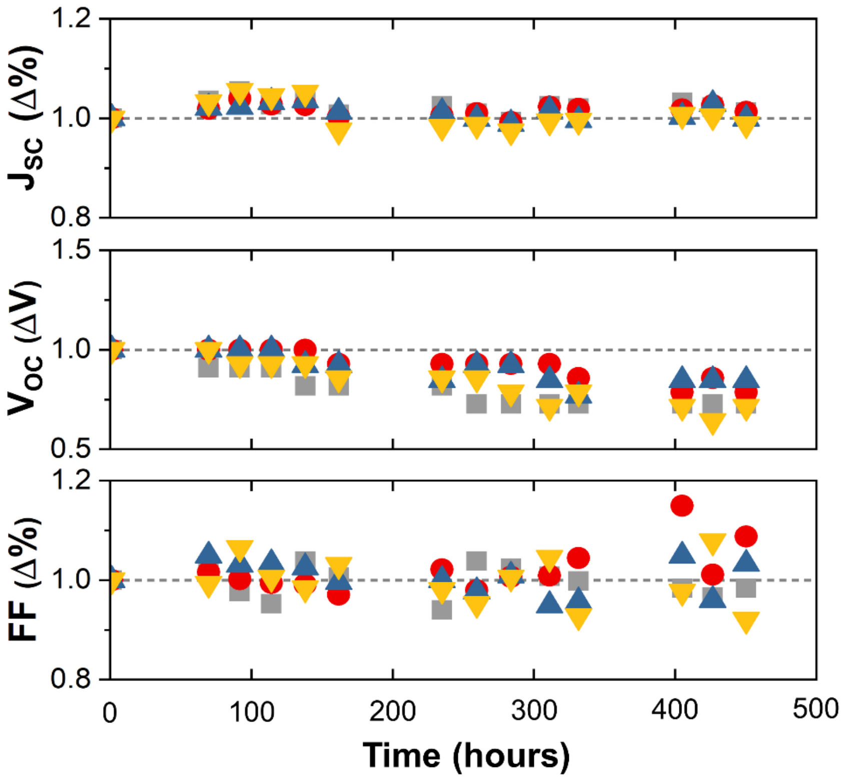

Figure S15. The evolution of J_{SC}, V_{OC}, and FF throughout the 450 hours of stability testing.
